# Supplementary material for: PUPAID: A R + ImageJ pipeline for thorough and semi-automated processing and analysis of multi-channel immunofluorescence data
Source: PLoS One. 2024 Sep 19;19(9):e0308970. doi: 10.1371/journal.pone.0308970 (PMC11412663; doi:10.1371/journal.pone.0308970)
Supplement: Attachment — Submitted filename: PUPAID_TransferRevision2_PLOS_One.docx [file pone.0308970.s001.docx]

**Reviewer #1**

*The main points that needed to be addressed from the previous review round were PUPAID's user friendliness and an appropriate quantitative benchmarking. Here is a response to these points, along with some additional comments:*

*User friendliness: The authors have implemented a GUI using R shiny which does improve PUPAID's user friendliness. However, instantiating the app still requires the user knowing how to execute a R command. While setting up R on a personal computer is beyond the scope of PUPAID’s manuscript, it would still be beneficial to provide a link to a broad audience introduction to R for new users at the beginning of the tutorial.*

We understand the reviewer’s point of view and added a new subsection in the part *1) Prerequisites* of the online tutorial, which basically introduces users to a reference resource for R beginners: *YaRrr! The Pirate’s Guide to R*. This online book is perfect to easily get knowledge about R programming language and the use of RStudio developing software.

*Benchmark: The authors have quantified PUPAID's segmentation performance using manual segmentation of cells in different confluence settings: low and high density. This performance has been put into context by comparing it to the segmentation offered by well-established tools such as Cellpose and Stardist. While certain setting combinations in PUPAID outperform both Cellpose and Stardist on some datasets, these tools seem to have been evaluated with their default settings. Although these algorithms could potentially be fine-tuned to achieve better performance, it would require either some coding knowledge and/or retraining models, which involves considerably more effort than evaluating different DoG sigma pairs. PUPAID's simplicity could therefore be presented as an advantage, as it allows users to explore different settings and select the best fit for their applications without extensive technical expertise.*

We thank the reviewer for his evaluation of our last revision. We indeed agree with him that we used pretrained models for StarDist and Cellpose segmentation methods. In the case of *StarDist*, we wanted to add that the pretrained model we used, called *Versatile (fluorescent nuclei)* was generated using a subset of the Data Science Bowl 2018 nuclei segmentation challenge dataset (<https://github.com/stardist/stardist>) from which the developers of *StarDist* selected only the images corresponding to 2D single channel with nuclei stained with fluorescent markers. As this dataset initially contains *“a large number of segmented nuclei images acquired under a broad variety of conditions and cell types, magnifications and imaging modalities”* (<https://paperswithcode.com/dataset/2018-data-science-bowl>), it is therefore intended to segment cells in the precise context we designed *PUPAID* for, hence the very good results we obtained with *StarDist* as compared to *PUPAID*, especially in the lowly-infiltrated tissue areas. This partly explains why we did not decide to generate another model more specific to our dataset. Plus, we also agree with the reviewer that it would have been very long and painful to construct another more fine-tuned model, especially because I am unfortunately not able to code in Python language, which is necessary for such operations with both *StarDist* and *Cellpose* implementations.

*Additional minor comments: The authors have successfully addressed the additional suggestions regarding CLAHE, DoG parameter explanation and suggestion, tabular data format, and the detail of the morphological metrics collected by PUPAID.*

*In conclusion, the manuscript has improved, and while state-of-the-art performances have not been challenged, PUPAID offers an accessible and efficient means of analysing multi-channel immunofluorescence data.*

**Reviewer #2**

*I would like to extend my gratitude to the authors for their diligent efforts in addressing the concerns I raised. However, after careful consideration, I regret to say that I am unable to recommend the manuscript for passage through the review process with the current version. Here are some major reaons:*

*- Figure 2: Missing parameters for running cellpose and stardist. Even though both cellpose and stardist provide pretrained model, it is always recommended to perform parameter tunings to achieve the best results. It is lack of rigour to compare a fine-tune workflow (PUPAID) to a pretrained naive model (CellPose/Stardist)*

We totally understand the reviewer’s point but we wanted to further explain ourselves as we do not fully agree with him. PUPAID is not a model-based segmentation tool, as it only relies on image processing coupled to the DoG method to extract cell contours. Thus, the only parameters that can be changed are notably incarnated by the σ values, which makes it easy for users to find the σ pair that produces the most relevant results for their own dataset. On the contrary, the use of model-based methods becomes very complicated when the models should be fine-tuned for the dataset of interest. In the case of *StarDist*, for instance, users can of course pretrain their own model, but to do this, they absolutely must master Python programming, and preferably have some basic notions about deep/machine learning and neural networks. These requirements are very unlikely to be met for most users. In my precise case, even if I know how to code in R, this is unfortunately not the case at all for Python. I was therefore unable to successfully train a fine-tuned model for my own dataset. The same applies for *Cellpose*. This is exactly why *PUPAID* is of interest, because it does not require users to have a deep knowledge of programming, especially since the latest revision of the manuscript and the software, as all can be launched through the R Shiny application. Still, tools such as *StarDist* and *Cellpose*, coupled with their pretrained models are of interest, because they provide precise and robust cell segmentation in a broad variety of contexts and datasets, without any user intervention. Of note, *StarDist* even proved to perform better than *PUPAID* for the lowly-infiltrated tissue regions, as the four cell segmentation quality metrics were higher than the ones obtained with *PUPAID*. Finally, we find the last sentence of the reviewer quite “unfair”, because the pretrained model we used for *StarDist* (called *Versatile (fluorescent nuclei)*) was generated using a subset of the Data Science Bowl 2018 nuclei segmentation challenge dataset (<https://github.com/stardist/stardist>) which contains *“a large number of segmented nuclei images acquired under a broad variety of conditions and cell types, magnifications and imaging modalities”* (<https://paperswithcode.com/dataset/2018-data-science-bowl>). This dataset was initially constructed in a way to force competitors to construct versatile cell segmentation methods that are intended to work for most images and datasets. Afterwards, the developers of *StarDist* only kept as training data for the *Versatile (fluorescent nuclei)* model the 2D single channel images showing cell nuclei stained with fluorescence markers. Therefore, this pretrained model is not “naïve” at all, as it was only trained on images that are accurately representative of the kind of modalities we actually want to analyse through *PUPAID*. Ultimately, we do not consider *PUPAID* as a “fine-tuned workflow”, as, again, only the choice of the σ pair for DoG-based cell segmentation can be changed, therefore drastically limiting the potential number of combinations users have to try in order to determine the optimal σ values for their own dataset.

*- Missing extra figure/screenshot/video recording about the R shiny App to execute the pipeline. Especially this is a shared concern about the user-friendlyness between reviewers.*

We totally agree with the reviewer that the tutorial is missing a better description of the R Shiny application. We indeed added to the tutorial a short video about how to launch the application. As pinpointed by the Reviewer #1, we also added another section to the tutorial which redirects users to a free and very useful online resource book for new/beginner R users.

*- Overall, it would be essential to adhere more closely to the standard academic writing style for greater professionalism, e.g. introducing FCS file format and interpretation of results in figure legend 3 are inappropriate; mentioned but not cited MiC imagej plugin properly; fuzzy claims in the manuscript, e.g. line 208, line 238 etc. I also kindly suggest refining the manuscript by minimizing the subjective descriptions in data interpretation. Clear presentation of results allows readers to discern differences without additional commentary, e.g. line 286, line 318-321. Last but not least, the evidences are not cited properly in the manuscript that supports the findings, e.g. which exact figure/subfigure that shows the "better performance"*

We understand the reviewer’s comment but wanted to clarify some elements. We agree that the introduction of FCS file format was not appropriate in the **Figure 3** legend, hence we moved it to the **Abstract** section. Nonetheless, we did not understand what the reviewer referred to when he wrote that *“the interpretation of the results in figure 3 are inappropriate”*. We simply described in the figure legend how FCS files are intended to be analysed (either through conventional flow/mass cytometry analysis software or through dedicated analysis pipelines, for instance written in R language). Using a set of plots obtained using data generated with *PUPAID*, we also described the gating strategy process which is a basic yet important way to analyse such data. We wanted the present actual results/plots to be as immersive as possible for the reader and the potential user, and especially for the biologists who are not very familiar with the flow/mass cytometry data analysis.

Regarding the *MiC* ImageJ plugin, their authors actually never published this plugin in a scientific journal, therefore making (sadly) impossible the fact to cite them or the plugin properly. Still, we added to the manuscript the GitHub link (<https://github.com/MultimodalImagingCenter/MiC>) from where it can be downloaded.

Regarding the sentences at line 208, we still consider it to correctly summarize the associated results, as this sentence is the conclusion of the **Figure S1**, which clearly showed that despite the fact that the percentage of detected objects with or without CLAHE was fairly identical with low variations, the measured objects without CLAHE showed significantly decreased cell segmentation metrics as compared to the CLAHE version, notably for the *Recall/sensitivity*, *Jaccard* and *F-measure* metrics between the 1:1.1 and 1:2 σ ratios. The *Precision* metric also showed a strong tendency to be lower when CLAHE is not used but it did not reach significance. Still, we modified the conclusion sentence to make our point clearer and more contextualized.

Unfortunately, we did not understand what the reviewer was referring to when mentioning the line 238, as the associated sentence simply described the methodology used throughout the **Figure 2** *(“Using the previously mentioned 200x200 pixels crops as well as the methodology described in the* ***Comparison of masks generated with different cell segmentation methods*** *section of the* ***Material and Methods S1****, we compared performance with different σ pairs against manual segmentation made by eye, but also against* StarDist *and* Cellpose *already published methods”*). Of note, we used the Manuscript Draft version generated by the PLOS Computational Biology submission system as reference for the line numbers.

Nevertheless, we thoroughly read the manuscript again and modified it to as close to the standard academic writing style as possible.

**Reviewer #3**

*I appreciate the authors putting in the much needed efforts of benchmarking their method against state-of-the-art image segmentation methods. However, I still predict that compared to these, given different and diverse datasets, these methods would be more robust especially since they, as PUPAID have sensitivity and probability parameters that the user can control to capture more cells.*

We understand the reviewer’s point of view, but still think that PUPAID could perform better at least in some cases, especially in high confluence settings. Our results show that PUPAID can outperform *StarDist* in such high confluence settings, and that *Cellpose* performs globally worse than PUPAID in all confluence settings. We wanted to clarify that PUPAID does not offer the possibility to tune any sensitivity or probability parameters, because PUPAID is only based on the DoG cell segmentation, therefore only needing a pair of σ values to determine cell contours. The quality metrics we showed in the manuscript were intended to compare other model-based methods such as *StarDist* and *Cellpose* with our own method. To analyse their datasets, users will only need to test several sigma pairs on representative images and then choose which ones lead to the best cell segmentation, which is easily done directly by eye. We wanted to offer another method to analyse this kind of immunofluorescence data without the use of model-based algorithms, which are indeed powerful, but also suffer from the quality of the training dataset. Plus, the fine-tuning of deep/machine-learning and neural networks models needs to master programming (here in Python), which is unlikely to be the case for the actual end users who only want to segment the cells on their images. Personally, I know how to code in R, but I am totally unable to transpose my knowledge in Python (or another language), which unfortunately prevents me to fine-tune these models.

*In addition the authors present this ImageJ-R hybrid as an advantage but I find it to be an unnecessary complication that is not justified. I would suggest to the authors to add PUPAID as a functionality to existing R packages and this way to offer the user the best of both worlds.*

We thank the reviewer for his remark. The possibility to include *PUPAID*’s workflow into another existing R package is actually pretty interesting but would require discussing with the respective developers of these packages, which could not be made at the moment of the writing of this point-by-point response. We also wanted to clarify that we implemented *PUPAID* the way it is because of the lack of R-written workflows to handle image processing as compared to the ones written in Java or Python. For developers, as me, who know how to code in R but not in Python nor C/C++ nor Java, biological image analysis can be very tricky and limited. Therefore, we decided to implement the backbone of the workflow in R, but to let the actual image handling to ImageJ, which is a very popular and powerful image analysis software. ImageJ is regularly updated, with a lot of plugins dedicated to a very broad range of tasks, which makes it very pertinent to analyse biological images. R only acts here as an interface between ImageJ and users, while offering supplemental functionalities, such as tiles auto-ordering and renaming, benchmarking of σ values, converting image analysis outputs to FCS files, etc.

*Once again I appreciate the authors efforts in making an open and accessible tool.*
