# Supplementary material for: PUPAID: A R + ImageJ pipeline for thorough and semi-automated processing and analysis of multi-channel immunofluorescence data
Source: PLoS One. 2024 Sep 19;19(9):e0308970. doi: 10.1371/journal.pone.0308970 (PMC11412663; doi:10.1371/journal.pone.0308970)
Supplement: Attachment — Submitted filename: Comment to authors PLOSone.docx [file pone.0308970.s002.docx]

Major observations

Thank you for your contribution with the development of PUPAID. While your work presents ground breaking potential, according to this version of the manuscript the real advantages appear to be primarily in high-density regions. Given the clinical expertise available among the authors, it would be highly beneficial to leverage this by providing detailed medical discussion.

From a reviewing point of view, it would help to be provided with a testing dataset, or at least to describe it, for comprehensive evaluation. Additionally, the manuscript lacks a thorough explanation of the benchmarking which appears a bit confusing. I think venturing in many comparisons, different in nature, is not simple to deal with. Improved communication on the quantitative methods used for benchmarking would significantly enhance the validation of PUPAID.

I also think the paper lacks demonstration of user-friendliness, with the language used resulting in an additional barrier. Including a figure or, preferably, a video of the interface would greatly enhance understanding and usability for readers.

Lastly, the manuscript's language is quite self-referential and would benefit from a more reader-centric approach, ensuring clarity and accessibility for a broader audience. I suggest giving more scientific context and sometimes guiding the reader via bullet points.

Minor changes:

Abstract

Line 58: Would be nice for reviewers to have test files to run the code

Introduction

Line 82-83: would appreciate if the author could elaborate on what is meant by “exponential increase of the complexity”. Example: what is the role of noise and how is it evolving through time. Also it is not very clear what is an “early lack of open-source methods”

Line 88: The learning curve of coding within publicly available tools can be steep, conversely I would not define programs like Image J cumbersome to master.

Line 94: would be interesting if the author could elaborate on the relevance of Doc Parra in assessing methodology performances. Additionally, please provide further evidence of the status quo claimed here.

Lines 110-112: quite a statement about performance, please provide quantitative insights.

Dependencies

Lines 124-126: could the author stress further the decision of relying on R rather than python packages. Are R packages flexible in terms of being integrated on cloud and how suitable are they for integration with AI workflows?

Pre-processing of raw data

Line 161: there are current efforts from the github community to manage such exotic formats https://github.com/cgohlke/czifile feel free to integrate in your script

Line 169: Is the “big idea” the innovation?

Line 170 and 180: It is a bit ambiguous to understand if the naming is either automatic or manual. Try to explain clearly and consider including an image solely dedicated to input data handling.

Processing of ROI

Lines 195-208: quite annoying to read through such a small amount of text calling back to so many supporting figures. Would it be possible to have a figure in main text or either organise the text differently? Benchmarking of either PUPAID-, manual- or other state-of-the-art methods-generatedcell segmentation

Line 237: the section here seems a mix of user instructions and benchmarking. By the way, consider including a user interaction section along with UI images, would probably be very beneficial to promote the adoption of the tool.

Line 247: provide quantitative details about the test datasets

Line 252-253: what is the error on the single metric you are using to claim significance? There are hints in the images but this information is a bit tough to retrieve on the spot.

Results

Line 319: how is the past application of a novel tool a result? Consider providing a clear explanation. You could refer to validation on published studies, and simply include the reference

Line 342: Again this sounds like a validation rather than result section.than result section.
