# Supplementary material for: PUPAID: A R + ImageJ pipeline for thorough and semi-automated processing and analysis of multi-channel immunofluorescence data
Source: PLoS One. 2024 Sep 19;19(9):e0308970. doi: 10.1371/journal.pone.0308970 (PMC11412663; doi:10.1371/journal.pone.0308970)
Supplement: Attachment — Submitted filename: PUPAID_PLOS_One_Revision.docx [file pone.0308970.s003.docx]

**Response to Editor and Reviewers**

*Thank you for submitting your manuscript to PLOS ONE. After careful consideration, we feel that it has merit but does not fully meet PLOS ONE’s publication criteria as it currently stands. Therefore, we invite you to submit a revised version of the manuscript that addresses the points raised during the review process.*

*The revisions were positive, highlighting the manuscript's potential impact in research fields. However, while your work presents groundbreaking potential, its real advantages seem to be primarily in high-density regions. Given the clinical expertise among the authors, a detailed medical discussion would be highly beneficial.*

*From a reviewer's perspective, providing or at least describing the testing dataset is essential for comprehensive evaluation. Additionally, the manuscript lacks a thorough explanation of the benchmarking, which appears somewhat confusing. Improved communication on the quantitative methods used for benchmarking would significantly enhance the validation of PUPAID.*

**Journal Requirements:**

*1. When submitting your revision, we need you to address these additional requirements.*

*Please ensure that your manuscript meets PLOS ONE's style requirements, including those for file naming. The PLOS ONE style templates can be found at* 
[*https://journals.plos.org/plosone/s/file?id=wjVg/PLOSOne_formatting_sample_main_body.pdf*](https://journals.plos.org/plosone/s/file?id=wjVg/PLOSOne_formatting_sample_main_body.pdf) *and* 
[*https://journals.plos.org/plosone/s/file?id=ba62/PLOSOne_formatting_sample_title_authors_affiliations.pdf*](https://journals.plos.org/plosone/s/file?id=ba62/PLOSOne_formatting_sample_title_authors_affiliations.pdf)

As requested by the Editor, we profoundly edited our manuscript to answer to the reviewers’ comments as well as to fully comply with the *PLOS One* journal requirements regarding the formatting of the manuscript. The revised version of the manuscript should fully embrace the journal requirements based on the two PDF resources the Editor provided to us.

*2. Thank you for uploading your study's underlying data set. Unfortunately, the repository you have noted in your Data Availability statement does not qualify as an acceptable data repository according to PLOS's standards.*

*At this time, please upload the minimal data set necessary to replicate your study's findings to a stable, public repository (such as figshare or Dryad) and provide us with the relevant URLs, DOIs, or accession numbers that may be used to access these data. For a list of recommended repositories and additional information on PLOS standards for data deposition, please see* [*https://journals.plos.org/plosone/s/recommended-repositories*](https://journals.plos.org/plosone/s/recommended-repositories)*.*

As requested by the Editor, and given the provided URL regarding the recommended repositories, we uploaded the example dataset on the Data Station Life Sciences repository hosted by the Data Archiving and Networked Services (DANS). The associated accession URL (<https://doi.org/10.17026/LS/7XQFAT>) was indeed added within the revised version of the manuscript.

*3. When completing the data availability statement of the submission form, you indicated that you will make your data available on acceptance. We strongly recommend all authors decide on a data sharing plan before acceptance, as the process can be lengthy and hold up publication timelines. Please note that, though access restrictions are acceptable now, your entire data will need to be made freely accessible if your manuscript is accepted for publication. This policy applies to all data except where public deposition would breach compliance with the protocol approved by your research ethics board. If you are unable to adhere to our open data policy, please kindly revise your statement to explain your reasoning and we will seek the editor's input on an exemption. Please be assured that, once you have provided your new statement, the assessment of your exemption will not hold up the peer review process.*

We thank the Editor for his remark. As requested in the previous point, we already uploaded the full example dataset on the Data Station Life Sciences repository hosted by the Data Archiving and Networked Services (DANS), which will be accessible *via* the following URL: <https://doi.org/10.17026/LS/7XQFAT>. Regarding the GitHub repository, all authors confirm that the code with be fully available if the manuscript is accepted for publication. Concretely, the developer and maintainer of *PUPAID* (Dr. Paul Régnier actually) only has to toggle a setting from “Private” to “Public” in his GitHub repository profile, which will instantly make the full code available to everyone. He again wanted to reassure the Editor that he will do this task without delay if the manuscript is accepted for publication.

*4. Please amend either the abstract on the online submission form (via Edit Submission) or the abstract in the manuscript so that they are identical.*

We wanted to apologize for this point, as this was not intentional at all. The corresponding author probably copied and pasted within the online submission form a version of the abstract which was not the very last one. We carefully checked both abstracts and made sure they were identical between the manuscript and the online submission system.

**Reviewers’s comments to the Author**

**Reviewer #1:**

*Thank you for your contribution with the development of PUPAID. While your work presents groundbreaking potential, according to this version of the manuscript the real advantages appear to be primarily in high-density regions. Given the clinical expertise available among the authors, it would be highly beneficial to leverage this by providing detailed medical discussion.*

We thank the reviewer for his very important remark. To discuss this interesting point, we added a new **Potential impact of PUPAID on the clinical and medical fields** section in the manuscript which details how *PUPAID* could be useful in the clinical and medical fields. We notably expose here tissues and their associated diseases for which immunofluorescence is widely used for both diagnostic and research purposes, and for which standardized and robust analysis methods could potentially help a lot to better understand the diseases’ pathophysiology, but also to accelerate the discovery of new biomarkers and further refine the classification of patients.

*From a reviewing point of view it would help to be provided with a testing dataset, or at least to describe it, for comprehensive evaluation. Additionally, the manuscript lacks a thorough explanation of the benchmarking which appears a bit confusing. I think venturing in many comparisons, different in nature, is not simple to deal with. Improved communication on the quantitative methods used for benchmarking would significantly enhance the validation of PUPAID.*

We thank the reviewer for his remark and made modifications to improve the points he highlighted. First, to ease readers understanding, we transferred and renamed the *Test dataset* section present in the **Supplemental Material and Methods** to a new *Example dataset for learning and test purposes* subsection in the main manuscript in the **Material and methods** section in order to present the example dataset, and notably what kind of organ/patient is the tissue from, how it was stained (technology, antibodies and Opal reagents) and how it was acquired and processed (instrument, software and demultiplexing step). We also indicated the new URL from which users can download the dataset.

We additionally moved every supplemental methods from the **Supplemental Material and Methods** to the main manuscript. This lengthens the manuscript a bit but allows to avoid switching between files for an explanation of a precise point. In a similar manner, we decided to integrate the old **Figure S1** as a new **Figure 2** in the main manuscript, to directly provide to readers the totality of the benchmarking data.

Second, we were unsure about what the reviewer specifically referred to when writing about the “benchmarking”, because the manuscript details two different ones. The first benchmarking was related to the CLAHE algorithm and helped to demonstrate that this step was pertinent and enhanced the subsequent cell segmentation through *PUPAID*. The second benchmarking was about the performance of *PUPAID* (with different σ ratios) as compared to manual cell segmentation but also to other well-established methods: *StarDist* and *Cellpose*. To improve the overall readability of the manuscript, we decided to edit both parts in the revised version of the manuscript, even if one was not directly targeted by the reviewer. In a sense, his remark tells us that the manuscript was probably not organized properly. To summarize, we transferred the **Comparison of masks generated with different cell segmentation methods** section originally present in the **Supplemental Material and Methods** to the main manuscript, in the **Benchmarking of the different cell segmentation approaches** subsection of the **Material and methods** section. We also extracted the CLAHE algorithm benchmarking part to a dedicated subsection between the **Processing of ROI** and **Benchmarking of either PUPAID-, manual- or other state-of-the-art methods-generated cell segmentation** subsections. This way, the methodology used for both benchmarking is totally visible and independent of the other sections, and the CLAHE-related section comes after the full description of the ROI processing. We believe this will improve the readability of the manuscript. These modifications indeed led to integrating the old **Figure S1** as the new **Figure 2**, whereas the old **Figure 2** is now the new **Figure 3** and the old **Figure 3** is now the new **Figure 4**.

*I also think the paper lacks demonstration of user-friendliness, with the language used resulting in an additional barrier. Including a figure or, preferably, a video of the interface would greatly enhance understanding and usability for readers.*

We greatly thank the reviewer for his very pertinent remark about the user-friendliness of *PUPAID*. Even if it was not as easy as one could think, we finally managed to generate a video of roughly 14 minutes which shows in live the full workflow of *PUPAID* applied on the example dataset. This video is accessible from the following YouTube link: <https://youtu.be/58Tm54OVP-g>.

*Lastly, the manuscript's language is quite self-referential and would benefit from a more reader-centric approach, ensuring clarity and accessibility for a broader audience. I suggest giving more scientific context and sometimes guiding the reader via bullet points.*

We greatly thank the reviewer for his constructive remark. To make our manuscript easier to read, we deeply modified its structure and content, as previously stated. First, the (now) full compliance with the *PLOS One* journal format helped a lot to restructure our work and our thoughts. Second, we rewrote entire sections of the manuscript to give more details (or less in some paragraphs) and to make a lot of sentences clearer. We also moved/switched full sentences/paragraphs to other (or even new) subsections to better structure the manuscript. Third, to enhance clarity and readability, we also put as main figures and methods the old supplemental materials originally submitted. This will ensure that readers will not have to permanently switch between documents when they read our manuscript. Fourth, we modified as much as we could the figures to make graphs and texts as big as possible to ease the reading. Fifth, we implemented a new subsection to add more scientific context regarding *PUPAID*’s utility and impact, notably in the medical field.

*Minor changes:*

*Abstract
Line 58: Would be nice for reviewers to have test files to run the code*

We thank the reviewer for his remark but wanted to clarify that test files (in this case, TIFF-formatted images containing multi-channel immunofluorescence data) were already available during the last submission of this manuscript, as mentioned in the last paragraph of the old **Availability and future directions** section of the originally submitted manuscript. If the reviewer encountered difficulty to access the files using the provided URL within the last weeks/months, we wanted to apologize for this issue, which was indeed not intentional. To address this problem in the long term, and in accordance with the Editor, we uploaded the test dataset to the Data Station Life Sciences repository hosted by the Data Archiving and Networked Services (DANS), where it should be available at all times. The test dataset will be totally accessible *via* the following URL: <https://doi.org/10.17026/LS/7XQFAT>.

*Introduction
Line 82-83: would appreciate if the author could elaborate on what is meant by “exponential increase of the complexity”. Example: what is the role of noise and how is it evolving through time. Also it is not very clear what is an “early lack of open-source methods”*

To be more precise, we wanted to underline the fact that over time, and especially during the two last decades, multiplex immunofluorescence underwent massive improvements together with a great diversification of methods, thus leading to the increase of the number of simultaneously acquired markers as well as the overall resolution and number of co-analyzed samples. We rephrased this sentence to be clearer and added references to emphasize on this point. We also removed the *early lack of open-source methods* part, because if it was true 10 to 20 years ago, it does not seem to be totally valid anymore, especially when we look at the plethora of analysis methods that were recently developed for this precise kind of purpose.

*Line 88: The learning curve of coding within publicly available tools can be steep, conversely I would not define programs like Image J cumbersome to master.*

We wanted to clarify what is said in this part of the manuscript. We did not mean to say that Image J is difficult to master. Actually, this software is very well programmed and organized, and offers the ability to quickly analyze heavy images with a broad variety of tools. However, if one needs to analyze dozens or hundreds of images using the same methodology in Image J, the need for automatization through macros and scripts (thus necessarily involving programming) is almost mandatory. To make this point of view clearer, we also rephrased this part.

*Line 94: would be interesting if the author could elaborate on the relevance of Doc Parra in assessing methodology performances. Additionally, please provide further evidence of the status quo claimed here.*

We thank the reviewer for his very constructive comment. Our initial sentence was meant to highlight the fact that the plethora of available analysis methods for immunofluorescence assays could potentially lead to the biased selection by researchers/scientists of methods that actually “produce” convenient results and thus avoid the “least performant” ones. We wanted to underline the notion that workflows which process data from A to Z (pre-processing, processing, normalization, analysis, results export, etc.) are one way to intent standardization of such analyses and avoid potential issues and biases. We thought this was relevant to write at first, but all your remarks taken as a whole finally lead us to doubt about the true relevance of this sentence in the introduction. In essence, our message is slightly suggested in the sentence *the vast majority of the available methods only seems to propose a given kind of analysis and almost never include actual pre-processing and processing of the raw images* written right before. In consequence, we thought it was better to remove the sentence related to Dr. Parra and thus rewrote the two last paragraphs of the introduction to make it clearer.

*Lines 110-112: quite a statement about performance, please provide quantitative insights.*

We thank the reviewer for his remark and added some quantitative insights in the last paragraph of the introduction but also added a new **Table 2** which summarizes the efficacy of *StarDist*, *Cellpose* and *PUPAID* (using its most balanced 1:1.4 σ ratio and an IoU threshold of 0.4) independently for each measurement (% of detected objects and each of the four mentioned metrics) and in average. This will help readers to have a simpler “take home” message regarding *PUPAID*’s efficacy as compared to other well-established cell segmentation methods.

*Dependencies*

*Lines 124-126: could the author stress further the decision of relying on R rather than python packages. Are R packages flexible in terms of being integrated on cloud and how suitable are they for integration with AI workflows?*

We thank the reviewer for his interesting remark. We wanted to clarify that the choice of the R language is mainly mediated by the fact that I master this language in a fluent way, which is absolutely not the case for Python. Actually, our laboratory is mainly oriented towards R instead of Python, because of the myriad of packages developed for the analysis of biological data in general, notably diffused through the CRAN, Bioconductor and also GitHub repositories. However, Python now offers a lot of analysis capabilities on a massive number of biological fields, which make them essentially comparable nowadays. Additionally, another argument in favor of R is that there are actually very few image analysis workflows in R, because they are mainly coded in Python or C/C++. We though that adding another R-based method would help scientists and researchers to better deploy the R language for image and immunofluorescence-based data.

Secondly, regarding the AI workflows integration, R already proposes a lot of AI-, deep-learning- and machine-learning-related packages such as *mlr*, *caret*, *keras*, *tidyverse*, *tensorflow*, *glmnet*, *chatAI4R, etc.* (for the most known and used). These packages currently offer similar functions as compared to their Python equivalent and perform equally. Additionally, a lot of online resources can easily complement this list and provide to developers solid basis in AI-based analyses: <https://cran.r-project.org/web/views/MachineLearning.html>, <https://www.coursera.org/articles/r-packages>, <https://www.tutorialspoint.com/7-best-r-packages-for-machine-learning>, <https://bradleyboehmke.github.io/HOML/>, <https://www.geeksforgeeks.org/introduction-to-machine-learning-in-r/>, <https://www.datacamp.com/tutorial/machine-learning-in-r>, <https://lgatto.github.io/IntroMachineLearningWithR/index.html>, <https://www.r-bloggers.com/2022/02/beginners-guide-to-machine-learning-in-r-with-step-by-step-tutorial/>, etc.

Finally, regarding the cloud integration part, even if R is not as popular as Python or other languages in this field, it is still perfectly possible to easily integrate the R environment within cloud computing resources, such as Amazon Web Services, Google Cloud or Microsoft Azure. The following resources detail the steps to follow in order to achieve such integration: <https://wiingy.com/learn/r-studio/connecting-rstudio-to-cloud-services/>, <https://aws.amazon.com/fr/blogs/opensource/getting-started-with-r-on-amazon-web-services/>, <https://cloud.google.com/architecture/data-science-with-r-on-gcp-eda>, <https://learn.microsoft.com/en-us/azure/machine-learning/how-to-r-interactive-development?view=azureml-api-2> and so on. As a real-life application, we at the lab already use every day an OpenStack-based computing node solution, which allows to launch Virtual Machines instances based on Linux allowing to run R code seamlessly with the benefit of our computing capabilities (hundreds of CPU cores with several TB of RAM and disk storage).

*Pre processing of raw data*

*Line 161: there are current efforts from the github community to manage such exotic formats* [*https://github.com/cgohlke/czifile*](https://github.com/cgohlke/czifile) *feel free to integrate in your script*

We thank the reviewer for this very pertinent resource, which allows to read CZI files using Python without the need for any software from Zeiss. Unfortunately, we doubt it could be directly integrated with *PUPAID* because it is Python-based, while *PUPAID* is R-based, which makes this package incompatible. However, we added its mention to the manuscript and rephrased the related sentence to give readers a broader measure of the tools available to read CZI files.

*Line 169: Is the “big idea” the innovation?*

We wanted to clarify what we meant by “big idea” here. We wanted to summarize what are the input files that *PUPAID* requires in order to perform its routine, which is incarnated by TIFF-formatted multi-channel images, either for a full ROI or for a batch of tiles. To avoid any misunderstanding or misinterpretation, we rephrased this sentence to make it clearer.

*Line 170 and 180: It is a bit ambiguous to understand if the naming is either automatic or manual. Try to explain clearly and consider including an image solely dedicated to input data handling.*

We thank the reviewer for his pertinent point we wanted to clarify what we meant. At line 170, we expose that *PUPAID* is able to automatically stitch any given number of tiles (in the case input data is made of tiles representing a full ROI). It is true that the “renaming”, even if actually made during the stitching step, is fully done by the script itself. So users do not need to manually rename anything during this step.

On the contrary, at line 180, we expose the next step of *PUPAID*, which occurs within ImageJ software through a macro script. In this precise step, it is required from users to give a name to each channel (layer) of the ROI, in order to further select which one to consider for the DoG algorithm application.

We acknowledge that these sentences could be subject to misunderstanding, and therefore rephrased these points to make them clearer. In accordance, we also slightly modified the **Figure 1**, which basically summarizes the whole *PUPAID* workflow.

*Processing of ROI*

*Lines 195-208: quite annoying to read through such a small amount of text calling back to so many supporting figures. Would it be possible to have a figure in main text or either organise the text differently?*

We again thank the reviewer for his very constructive remark. We agree that the position of the supporting figure was indeed not optimal at all. To complement what was previously mentioned in this point-by-point response, we took advantage of the whole manuscript restructuring to switch all the supporting methods and figures to the main text. Although the manuscript is now a bit longer, it does not need any more to switch between documents. Finally, we also reworked the new **Figure 2** and **Figure 3** to have their displayed graphics and texts as big as possible to ease reading.

*Benchmarking of either PUPAID-, manual- or other state-of-the-art methods-generated
cell segmentation*

*Line 237: the section here seems a mix of user instructions and benchmarking. By the way, consider including a user interaction section along with UI images, would probably be very beneficial to promote the adoption of the tool.*

We thank the reviewer for his remark. We agree that the lines 237-241 were a mix of user instructions and benchmarking, which can be confusing. We removed these lines, and decided to complete, as suggested, the manuscript with a video showing a full *PUPAID* analysis workflow. This will certainly help readers to better appreciate how to use our tool. Although the creation of a new figure with UI images could have been possible, it would have been huge due to the numerous steps and elements to show, which rather oriented us towards the creation of a video.

*Line 247: provide quantitative details about the test datasets*

We wanted to clarify that the quantitative details about the test dataset are fully available in the new **Figure 3B-D**. The new **Figure 3A** only shows a representative example of how each compared cell segmentation method performs (one example for a single HD region, and another one for a single LD region), but it effectively does not provide any quantitative insight, which are of course detailed right after in the figure. To avoid any confusion, we indeed edited the associated part in the manuscript.

*Line 252-253: what is the error on the single metric you are using to claim significance? There are hints in the images but this information is a bit tough to retrieve on the spot.*

We thank the reviewer for his remark and wanted to clarify that we used the Standard Error of the Mean (SEM) as error metric, which was already written in the associated figure legend. Furthermore, as previously mentioned in this point-by-point response, we added a new **Table 2** which summarizes the mean of each measured metric to compare the different cell segmentation methods. For the sake of readability, we did not find useful to include the SEM in this new table, but they can be provided anytime if the reviewer finds it pertinent.

*Results*

*Line 319: how is the past application of a novel tool a result? Consider providing a clear explanation. You could refer to validation on published studies, and simply include the reference*

We greatly thank the reviewer for his very pertinent remark. We wanted to clarify the process which led to the existence of this section and its content. It is actually related to the initial submission process of this manuscript to *PLOS Computational Biology*, which specifies in its submission guidelines related to the description of software (<https://journals.plos.org/ploscompbiol/s/submission-guidelines#loc-software-submissions>) that manuscripts should contain a *Results* section which is intended to show *Examples of biological problems solved using the software, including results obtained with the deposited test data and associated parameters*. The *PLOS Computational Biology* journal unfortunately refused our manuscript but decided to allow a direct transfer to *PLOS One* because they found it interesting and more suitable for this journal. Therefore, we directly proceeded with the submission to *PLOS One* without changing the format of the manuscript, hence explaining why it does not comply for now with the formatting imposed by *PLOS One*. To comply with this format and to avoid any misunderstanding regarding the remark of the reviewer, we renamed the *Results* section to a more appropriate one: *Application of* PUPAID *to already published studies*.

*Line 342: Again this sounds like a validation rather than result section.*

As the two last points of the reviewer are indeed referring to the same subject, please refer to our response right before this one.

**Reviewer #2:**

*The manuscript PONE-D-24-13313 by P. Régnier and colleagues presents PUPAID, a workflow developed in R and ImageJ for semi-automated processing and analysis of multi-channel immunofluorescence data. They explain its workflow, which is user-friendly by incorporating an optional R Shiny-based interactive application for those not proficient in R. PUPAID performance is validated on few datasets and compared with state-of-the-art methods like StarDist or Cellpose, identifying conditions where it outperforms them, especially in dense areas. It exports single-cell data as FCS files, compatible with various cytometry analysis software. PUPAID is available as a GPLv3-licensed R package on GitHub. The work is well presented and with convincing experimental results and it is well written. The installation and usage is well documented and reproducible.*

*I support the publication of the manuscript without additional changes.*

We greatly thank Dr. Gavryusev for his very positive review of our manuscript and software. We made our best to improve them as much as possible and are very glad these were so well received. Thank you again for your time.
